# Freund's vaccine adjuvant promotes Her2/Neu breast cancer

**DOI:** 10.1186/1471-2407-9-19

**Published:** 2009-01-14

**Authors:** Michelle S Cotroneo, Jill D Haag, Nicholas R Stapel, Jordy L Waller, Stephan Woditschka, Michael N Gould

**Affiliations:** 1 McArdle Laboratory for Cancer Research, Department of Oncology, University of Wisconsin-Madison, 1400 University Avenue Madison, Madison, WI, USA

## Abstract

**Background:**

Inflammation has been linked to the etiology of many organ-specific cancers. Indirect evidence suggests a possible role for inflammation in breast cancer. We investigated whether the systemic inflammation induced by Freund's adjuvant (FA) promotes mammary carcinogenesis in a rat model in which cancer is induced by the *neu *oncogene.

**Methods:**

The effects of FA on hyperplastic mammary lesions and mammary carcinomas were determined in a *neu*-induced rat model. The inflammatory response to FA treatment was gauged by measuring acute phase serum haptoglobin. In addition, changes in cell proliferation and apoptosis following FA treatment were assessed.

**Results:**

Rats receiving FA developed twice the number of mammary carcinomas as controls. Systemic inflammation following FA treatment is chronic, as shown by a doubling of the levels of the serum biomarker, haptoglobin, 15 days following initial treatment. We also show that this systemic inflammation is associated with the increased growth of hyperplastic mammary lesions. This increased growth results from a higher rate of cellular proliferation in the absence of changes in apoptosis.

**Conclusion:**

Our data suggests that systemic inflammation induced by Freund's adjuvant (FA) promotes mammary carcinogenesis. It will be important to determine whether adjuvants currently used in human vaccines also promote breast cancer.

## Background

The etiology of breast cancer involves the interaction of inherited risk with environmental exposure. Approximately 30% of breast cancer risk is inherited [1], the majority of which is controlled by modifier genetic elements individually having low genetic penetrance, but high population frequencies [2]. Our knowledge of environmental factors that modulate breast cancer risk is quite incomplete. The best documented association is for ionizing radiation, which carries the highest risk for breast cancer when exposure occurs prior to adulthood [3]. Other known promoters of breast cancer include exogenously administered hormonal replacement therapies [4].

Chronic inflammation arising from a variety of environmental and infectious sources is associated with promotion of many cancer types [5]. Evidence for chronic inflammation in breast cancer etiology is supported by experimental [6] and epidemiological studies suggesting that anti-inflammatory drugs can help prevent breast cancer [for review, see ref. [7]]. It thus can be hypothesized that agents which induce a systemic inflammatory response might increase breast cancer risk. The age window of maximum susceptibility to breast cancer initiation by environmental radiation occurs before age 20 [3]. During this period of life, individuals are exposed to vaccine adjuvants, which bolster the immune response to antigens and often cause local and systemic inflammatory responses. In order to begin to evaluate a role for systemic inflammation in the etiology of breast cancer, Freund's adjuvant (FA), which is known to induce both local and systemic inflammation [8] was evaluated for its ability to modulate breast cancer development in a rat model in which cancer is induced by the *neu *oncogene.

## Methods

### Adjuvant treatments

All animal study protocols were approved by the University of Wisconsin Institutional Animal Care and Use Committee (IACUC). Virgin Wistar-Furth (WF) rats were obtained from Harlan Sprague-Dawley (Indianapolis, IN), housed in our facility on a 12 hour light/dark cycle and given free access to standard lab chow. For all studies, euthanasia was carried out using CO_2 _inhalation. Rats were injected subcutaneously in the dorsal region with Freund's complete adjuvant (CFA) or Freund's incomplete adjuvant (IFA) (Sigma-Aldrich) suspended in sterile 0.9% PBS. Three treatment protocols were used: early schedule, late schedule and single dose. Rats on the early schedule protocol received CFA (0.5 ml/kg body weight) at 5 weeks of age and IFA (0.5 ml/kg body weight) at 7, 9 and 13 weeks of age. Rats on the late schedule were injected with CFA at 10 weeks of age and IFA at 12 and 14 weeks. All control groups received an equivalent volume of saline. In the single dose model, CFA or saline was injected into 10 week old rats at a concentration of 0.5 ml/kg body weight, and rats were euthanized 1 (n = 5/group) or 15 days (n = 6/group) after injection.

### Mammary tumorigenesis

Virgin WF female rats were injected with adjuvant using the early (n = 15) and late (n = 15) treatment protocols, as described above. Controls (n = 14) received saline using the early schedule. Mammary carcinomas were induced by intraductal infusion of the pJR*neu *vector into the mammary epithelium (12 glands/rat) of 8 week old rats, using a viral titer of 1 × 10^5 ^CFU/ml. The generation and mammary intraductal infusion of the pJR*neu *retroviral vector has been previously described [9]. Rats were palpated weekly for the presence of mammary carcinomas starting at 9 weeks of age and removed from the study at 17 weeks of age (9 weeks post-infusion). Mammary carcinoma multiplicity was recorded for each rat by counting the mammary carcinomas greater than 3 mm in diameter.

To characterize hyperplastic mammary lesions in FA-treated rats, the fourth mammary glands of rats on the early treatment protocol (n = 20–21/group) were infused with the pJR*neu *vector at a viral titer (1 × 10^7 ^CFU/ml). This higher titer was previously determined to provide sufficient statistical power, while minimizing the number of required animals [10]. Rats were removed from the study 15 days post-infusion, and one of the infused mammary glands per rat was whole mounted and stained with aluminum carmine as previously described [11]. Hyperplastic mammary lesions were identified from the whole mounts by their size and counted using 20× light microscopy to determine lesion multiplicity. Each stained mammary gland was photographed using a SONY Cybershot digital camera using 1.3× optical zoom. The area of each hyperplastic lesion, mammary gland and lymph nodes was determined from digital photographs using ImageJ (Scion Corp.), available from the NIH website http://rsb.info.nih.gov/ij/. Two methods were used for statistical comparison of area measurements from the hyperplastic mammary lesions. First, the average lesion size was determined for each treatment (n = 179 lesions from controls and 258 from adjuvant-treated rats). Secondly, the total lesion area was determined for each mammary gland (n = 20–21/group) and normalized to its respective mammary gland area. The normalized values were then combined for each treatment group for statistical analysis. The contralateral mammary glands (n = 10/group) were fixed in 10% neutral buffered formalin and stained with hematoxylin and eosin for histopathological analysis.

### Evaluation of inflammatory response to adjuvant

Acute phase serum haptoglobin levels were measured in rats injected with CFA or saline using the single dose protocol, as described above. Following euthanasia, blood was collected from the aorta using a syringe and an 18 gauge needle and allowed to clot. Serum was collected after centrifugation, aliquotted and stored at -80°C. Haptoglobin levels were determined using the Tri-Delta Diagnostics phase haptoglobin colorimetric assay, according to the manufacturers' instructions. All samples, including standards, were run in triplicate.

### Immunohistochemical analysis for cell proliferation/apoptosis

Mammary epithelial cell proliferation was evaluated by immunohistochemistry for Ki67 in non-infused mammary glands from rats on the early adjuvant schedule, and from hyperplastic mammary lesions in virally infused rats given early treatment of either adjuvant or saline. All rats were sacrificed at 10 weeks of age. All reagents for Ki67 staining were obtained from Vector Laboratories unless otherwise stated. Sections were immersed in antigen retrieval solution at 95°C for 20 minutes following deparaffinization in xylene and rehydration in decreasing concentrations of ethanol. Endogenous peroxide activity was inhibited by incubation with 3% H_2_0_2_, and nonspecific antibody binding was prevented by treating with 10% normal goat serum and avidin/biotin blocking solutions. Sections were incubated at 4°C overnight with a 1/500 dilution of mouse monoclonal anti-Ki67 antibody. After washing with PBS, sections were then incubated with a 1/200 dilution of biotinylated anti-mouse IgG. Detection of Ki67 antibody binding was done using a Vectastain ABC kit with DAB chromogen (BD Biosciences Pharmingen).

The fraction of proliferating epithelial cells in mammary structures was determined by counting Ki67+ and Ki67- cells magnified 200× with a light microscope (Olympus BX50) and photographed using a digital camera with Magnafire image capture software (Optronics). In the mammary glands of rats given adjuvant (n = 8) or saline (n = 7) without retroviral infusion, terminal end buds (TEB) and ducts/alveolar structures were analyzed as separate compartments. All TEBs present in each section (80 control and 47 adjuvant-treated) were analyzed. For other ductal and alveolar structures, all epithelial cells present in 8–10 random fields (200×) per slide (n = 6/group) were analyzed. For rats infused with *neu*, epithelial cell counts were done from digital photos taken at 40× magnification of the sections of the hyperplastic mammary lesions (n = 10 rats/group). All lesions with adequate staining were analyzed, for a total of 29 controls and 22 adjuvant-treated.

Apoptosis was assessed in hyperplastic mammary lesions by labeling of 5' ends of fragmented DNA with terminal deoxynucleotidyl transferase (TdT) enzyme using a TdT-FragEL kit, following the manufacturers' instructions (Oncogene/Invitrogen). The fraction of apoptotic epithelial cells in hyperplastic mammary lesions was determined as described for Ki67.

### Statistics

All statistical analyses were done using StatView (SAS, Cary, NC). Mammary carcinoma multiplicity was tested using One-Way ANOVA with Bonferroni-Dunn multiple comparison. Serum haptoglobin, cell proliferation/apoptosis immunohistochemistry and hyperplastic mammary lesion multiplicity were analyzed by unpaired *t *test and nonparametric (Mann Whitney) testing, where appropriate. Statistical analysis of serum haptoglobin values was done separately for the two time points (1 day and 15 days), since each had its own control group. Area measurements (mammary gland, lymph node and hyperplastic mammary lesions) were analyzed by unpaired *t *test both as raw data and following log transformation to more closely approximate a normal distribution. Since the conclusions were the same for raw and log transformed data, we present the results for the raw data.

## Results

### Adjuvant treatment increases incidence of mammary carcinomas following infusion of activated *neu*

**Figure 1:**
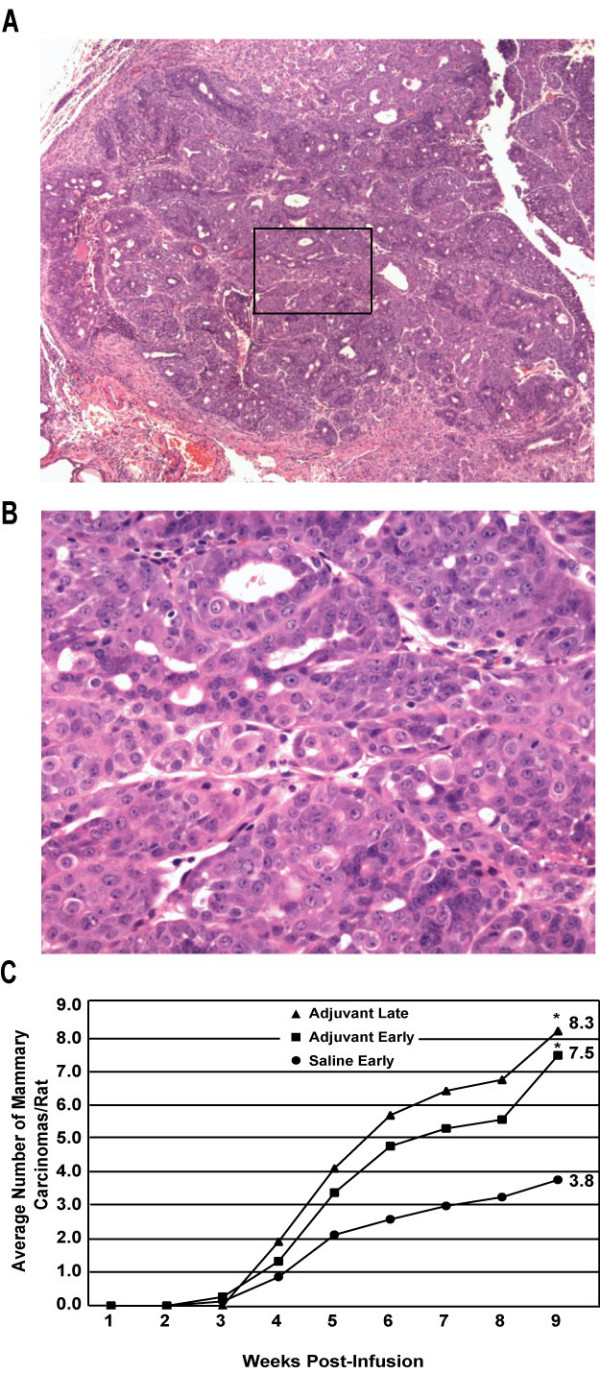
**Adjuvant treatment increases *neu*-induced mammary carcinoma multiplicity**. **A**. Representative histologic section of mammary carcinoma 12 weeks post-infusion, hematoxylin and eosin (H&E) staining, 40× magnification. **B**. 200× magnification of area defined by box in panel **A**. **C**. Rats were injected subcutaneously with 0.5 ml/kg body weight Freund's complete adjuvant (CFA) at 5 (early schedule, n = 15) or 10 (late schedule, n = 15) weeks of age. Booster injections of Freund's incomplete adjuvant (IFA) were given at 7, 9, and 13 (early schedule) or 12 and 14 (late schedule) weeks of age. Controls received saline on the early schedule (n = 14). Rats were palpated weekly for carcinoma development following the infusion of the pJR*neu *retroviral vector into the mammary ducts at 8 weeks of age and euthanized 9 weeks post-infusion. *P < 0.05 compared with controls, One-Way ANOVA with Bonferroni-Dunn multiple comparisons.

Direct transfer of the *neu *oncogene into the mammary ductal cells results in clonal development of mammary carcinomas (Figure 1A and 1B) [12,13]. This model thus provides a unique system to assess the effects of chronic inflammation on breast cancer development. Treatment of rats with adjuvant using either the early or late schedule resulted in significantly increased mammary tumor multiplicity following infusion with *neu *compared with saline controls (Figure 1C). At 9 weeks after ductal infusion, adjuvant-treated rats had approximately twice the number of carcinomas than did the controls (P < 0.05). There were no histopathological differences between mammary carcinomas arising in the various treatment groups.

### Adjuvant treatment induces a systemic and chronic inflammatory response and increases mammary epithelial cell proliferation

**Figure 2:**
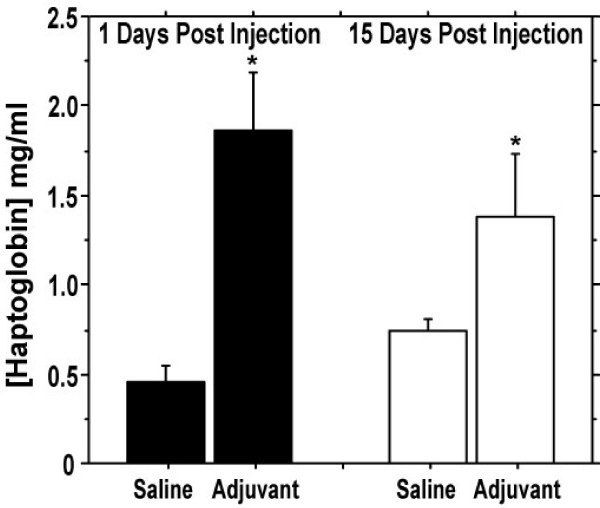
**Adjuvant treatment acutely and persistently elevates circulating haptoglobin concentrations in the serum**. Rats were given a single subcutaneous injection of CFA, (0.5 ml/kg body weight) and sacrificed 1 (n = 5/group) or 15 (n = 6/group) days later. Error bars represent +1 SD. *P < 0.01, Mann-Whitney Rank test.

To assess for evidence of chronic systemic inflammation, we measured serum haptoglobin concentrations [8]. Haptoglobin was elevated (~4-fold) 24 hours after a single injection of CFA and was approximately 2-fold higher than controls 15 days after injection (P < 0.01) (Figure 2).

Since a systemic inflammatory response was present, we hypothesized that the products of inflammation, such as cytokines and growth factors, may affect proliferation in the mammary epithelium. In adjuvant-treated rats on the early schedule protocol without retroviral infusion, staining for Ki67 showed a modest but statistically significant increase in proliferation in the mammary gland epithelium compared to controls (P < 0.05) (Table 1). This was true for both epithelial cells in terminal end buds and for ductal/alveolar structures. Interestingly, we did not observe an increase in inflammatory cells within the mammary parenchyma of adjuvant-only treated rats.

**Table 1:** Analysis of epithelial cell proliferation in mammary glands and *neu*-induced hyperplastic mammary lesions by Ki67 immunohistochemistry

	Percentage of Proliferating Cells ± SD		
	
Treatment	Terminal End Buds	Ductal/Alveolar Structures	Hyperplastic Mammary Lesions
Saline	56% ± 1	13% ± 1	None
Adjuvant	61% ± 1*	17% ± 1*	None
Saline + *neu*	N.A.	N.A.	41% ± 2
Adjuvant + *neu*	N.A.	N.A.	55% ± 2**

N.A.= not analyzed

*P < 0.05, **P < 0.001 (Mann-Whitney Rank test)

### Adjuvant treatment given prior to infusion of *neu *oncogene increases the size of hyperplastic mammary lesions by increasing cell proliferation

**Figure 3:**
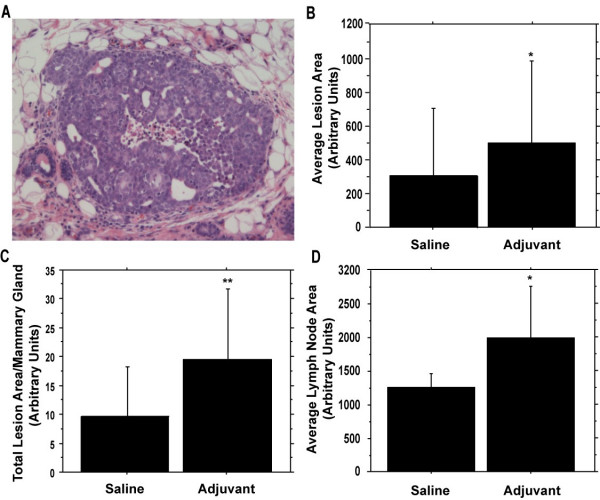
**Adjuvant treatment increases the size of hyperplastic mammary lesions 15 days following *neu *infusion and results in localized lymphadenopathy**. Rats were given 0.5 ml/kg body weight CFA at 5 weeks of age and booster injections IFA at 7 and 9 weeks of age (n = 21). Controls received saline (n = 20). Infusion of the *neu *oncogene retroviral vector into the fourth mammary gland was done at 8 weeks of age. **A**. H&E staining of a representative hyperplastic mammary lesion (100× magnification). **B**. Average lesion area determined for each treatment (n = 179 lesions from controls and 258 from adjuvant-treated rats). **C**. Total lesion area per gland, normalized to individual mammary gland area (n = 20–21/group). **D**. Average area of the intramammary lymph nodes from infused glands (n = 20–21/group). Error bars represent +1 SD. *P < 0.001; **P < 0.01, *t *test.

We characterized the proliferative effects of Freund's adjuvant (early schedule treatment) on hyperplastic mammary lesions by analyzing mammary glands 15 days after *neu *infusion. This time point precedes the formation of palpable tumors, which occurs 3–4 weeks post-infusion. A histological section of a typical hyperplastic mammary lesion is shown in Figure 3A. Lesion multiplicity was higher in rats given adjuvant than in saline controls (12.3 versus 8.9 respectively) but did not differ statistically (P = 0.13, unpaired *t *test).

The average size of the hyperplastic mammary lesions determined from area measurements was significantly higher (1.6-fold) in adjuvant-treated rats when compared to controls (P < 0.001) (Figure 3B). In addition, the total lesion area per mammary gland (normalized to mammary gland area) was higher (~2-fold) in adjuvant-treated rats versus controls (P < 0.01) (Figure 3C). The larger lesion size led us to evaluate cell proliferation and apoptosis using immunohistochemistry. The epithelial cells within the hyperplastic mammary lesions were significantly more proliferative in adjuvant-treated rats, as determined by the percentage of Ki67+ cells, than in controls (P < 0.001) (Table 1). The apoptotic fraction of cells in the hyperplastic mammary lesions was low, and did not differ significantly between saline (1.2%) and adjuvant (1.9%) groups (P = 0.31, Mann-Whitney Rank test).

In addition to adjuvant-induced proliferation, we also observed localized lymphadenopathy 15 days after *neu *infusion in adjuvant-treated rats. The average area of lymph nodes residing in the fourth mammary gland was significantly higher (1.6-fold) in adjuvant-treated rats, as compared to controls (P < 0.001) (Figure 3D).

## Discussion

The role of local inflammation in the colon [14,15] and prostate [16] in the etiology of these organ-specific cancers is well supported in humans. The role of inflammation in other cancer sites has been hypothesized. Here, we used a rat model to show that systemic inflammation induced by Freund's adjuvant (FA) can promote the development of mammary carcinomas induced by *neu*, a gene associated with breast cancer. This tumorigenic response to FA was similar if given before or after the initiation of carcinogenesis. This indicates that the effect of FA is not due to altered infection efficiency of the *neu *viral vector. FA administration produces a chronic state of systemic inflammation, as determined by the serum haptoglobin, an excellent cross-species marker for inflammation [8]. This finding is also supported by the observation of localized mammary lymphadenopathy two weeks following FA exposure.

Treatment with FA resulted in an increase in normal mammary cell proliferation. Two weeks after initiating mammary cancer with *neu*, many hyperplastic lesions are found in the mammary gland, ranging in histopathological progression from intraductal hyperplasia to ductal carcinoma *in situ *(DCIS) [9]. The main effect of FA-induced inflammation on these lesions was to increase the proliferative rate of these cells without significantly modifying the apoptotic index. Thus, FA administration likely promotes the growth of hyperplastic mammary lesions, which may lead to an increase in carcinoma formation. The presence of chronic inflammation suggests that the immune system may mediate mammary lesion proliferation through systemic or local regulation of cytokines and/or growth factors.

## Conclusion

It is important to point out that FA is a very strong adjuvant, and is not used in humans. However, weaker adjuvants, such as aluminum salts, are routinely used in most human vaccines for similar purposes [17]. In addition, much work, including clinical trials, is now underway to develop more potent and effective adjuvants for human use [18]. Such adjuvants would have the potential to both focus and intensify the immune response and also to reduce the amount of vaccine antigen needed per vaccination. Since many vaccinations are given in childhood and adolescence, the times of greatest susceptibility to breast cancer initiation [3], it will be important to evaluate the safety of both currently used and new vaccine adjuvants in experimental models and epidemiologic studies that focus on breast cancer etiology.

## Abbreviations

FA: Freund's adjuvant; IACUC: Institutional Animal Care and Use Committee; WF: Wistar-Furth; CFA: Freund's complete adjuvant; IFA: Freund's incomplete adjuvant; PBS: phosphate buffered saline; NIH: National Institutes of Health; ABC: avidin: biotinylated enzyme complex; DAB: diaminobenzidine; TEB: terminal end buds; TdT: terminal deoxynucleotidyl transferase; ANOVA: analysis of variance; DCIS: ductal carcinoma *in situ*

## Competing interests

The authors have neither financial nor non-financial competing interests to declare in relation to this manuscript.

## Authors' contributions

MSC co-designed, carried out, and analyzed resulting data from animal experiments generating hyperplastic mammary lesions and co-drafted and edited the manuscript.

JDH co-designed and helped performing the animal experiments with mammary carcinomas as scored endpoints and revised the manuscript. NRS co-designed and carried out the animal experiments with mammary carcinomas, generated serum biomarker data and performed the statistical analysis supporting these data. JW generated the retrovirus used in the rat infusion experiments and co-designed and carried out the animal experiments using mammary carcinomas as scored endpoints. SW co-designed and carried out the animal experiment generating hyperplastic mammary lesions and revised the manuscript. MNG conceived, co-designed, and coordinated all animal experiments and revised the manuscript. All authors read and approved the final manuscript.

## Pre-publication history

The pre-publication history for this paper can be accessed here:

http://www.biomedcentral.com/1471-2407/9/19/prepub
